# Risk Factors Associated with Survival of Pulmonary Tuberculosis

**Published:** 2018-07

**Authors:** Mehdi KAZEMPOUR DIZAJI, Anoshirvan KAZEMNEJAD, Payam TABARSI, Farid ZAYERI

**Affiliations:** 1. Dept. of Biostatistics, Faculty of Medical Sciences, Tarbiat Modares University, Tehran, Iran; 2. Mycobacteriology Research Center, National Research Institute for Tuberculosis and Lung Diseases (NRITLD), Shahid Beheshti University of Medical Sciences, Tehran, Iran; 3. Clinical Tuberculosis and Epidemiology Research Center, National Research Institute for Tuberculosis and Lung Diseases (NRITLD), Shahid Beheshti University of Medical Sciences, Tehran, Iran; 4. Dept. of Biostatistics, Faculty of Paramedical Sciences, Shahid Beheshti University of Medical Sciences, Tehran, Iran

**Keywords:** Tuberculosis, Generalized gamma regression model, Pulmonary TB

## Abstract

**Background:**

We conducted this study among adults with pulmonary tuberculosis (TB) who received treatment, in order to determine the risk factors associated with survival of during treatments.

**Methods:**

A retrospective cohort study was conducted from 2005–2015 with newly registered TB patients in the Hospital of Masih Daneshvari Doctor, Tehran, Iran. Overall, 5313 patients met our study’s cohort definition, but the analysis was performed on 2299 patients (43.2%) who had a correct address and they could be traced-out by the Medical – registry. Time in days was used in survival model and patients who were still alive (until last follow-up date) considered as censored. To study the effect of risk factors on patients’ survival, the generalized gamma regression model was used.

**Results:**

Based on the results of univariate analysis, gender (RR=2 (95% CI: 1.1–3.7), high school education (Relative Risk: RR=0.3 (95% CI: 0.2–0.7), higher education (RR=0.3 (95% CI: 0.1–0.9), smoker (RR=2.5 (95% CI: 1.4–4.2), drug user (RR=2.4 (95% CI: 1.4–4), TB contact (RR=0.5 (95% CI: 0.3–0.8) and HIV positive (RR=4 (95% CI: 1.7–9.2) affected patients’ survival. Moreover, the results of multivariate analysis showed that, gender (RR=5.5 (95% CI: 2.2–13.5), age (RR=1.1 (95% CI: 1–1.1), adverse drug effect (RR=2.5 (95% CI: 1.2–5.4), smoker (RR=3.3 (95% CI: 1.2–9.4), TB contact (RR=0.2 (95% CI: 0.1–0.5), diabetic mellitus (RR=3 (95% CI: 1–8.3), HIV positive (RR=26 (95% CI: 4.6–145.9) and comorbidities (RR=4.9 (95% CI: 2–11.6) were identified as factors affecting patients’ survival.

**Conclusion:**

Our data indicated associated risk factors in TB mortality and could suggest way to progressing national tuberculosis program (NTP) for predicating and plan for effective interventional strategies.

## Introduction

Despite effective treatment strategies, tuberculosis (TB) still remains a major health problem. It infects millions of people and ranks as the second leading cause of death from infectious diseases, after human immunodeficiency virus (HIV). In 2013, there were an estimated 12 million TB cases, including 8.6 million new cases, and 1.5 million fatal cases (1).

Clinical trials in developed countries revealed that 6.6% of TB patients died during or after therapy, whereas, in underdeveloped and developing countries the death rate was higher due to multidrug-resistant TB (MDR) and prevalence of HIV infection (2). The TB death rates globally are variable (7% to 35%) and reported to be different for each county (3, 4). Researches attributed TB mortality could consider to either TB or to unrelated diseases such as cardiovascular diseases, diabetes mellitus, HIV, cancers and drug toxicity (5).

Iran is a country with medium incidence of TB (21 per 100000 individuals) and low rate of TB mortality (4%) (6). Recently, few independent researchers showed a higher mortality rate (3.15% to 10%) for Iran cross-border provinces (7). At present we have no explanation for these discrepancies, but the proximity of Iran to the countries like Pakistan, Afghanistan, Iraq, and Azerbaijan might increase the risk of TB deaths in such area. Unfortunately, the lack of uniformity and accurate registration system, make the judgment more difficult in this area. Thus, the novelty and importance of the current study were to explore risks factors related to the increased mortality of TB especially pulmonary TB with time in population. Not much information available on this subject and we are the first groups for following up for 10 years.

There are various parametric models to assess the effects of risk factors on survival of pulmonary tuberculosis patients, including exponential, Weibull, log-normal, log-logistic, and generalized gamma regression models (8–12). The generalized gamma distribution showed better fit than the others, parametric models. Therefore, in this study to assess the risk factors for pulmonary tuberculosis, generalized gamma regression model has been used.

In the present study, we aimed to evaluate the causes of TB death during treatments of pulmonary TB who referred to National Referral TB Hospital in Tehran from all over the country. The information was not only collected through medical registry, but also by interviewing the family. For our knowledge, the current study is the first report of TB deaths in a longer period, i.e., 10 yr. The result of this study may help to identify the associated risk factors in TB mortality and could suggest to the national tuberculosis program (NTP) to predicate and plan for effective interventional strategies.

## Materials and Methods

### Study population

A retrospective cohort study was conducted from 2005–2015 with newly registered TB patients referred to the National Referral Center for Tuberculosis at Dr. Masih Daneshvari Hospital Tehran, Iran. In total, 5313 patients met our study’s cohort definition, but the analysis was performed on 2299 patients (43.2%) who had a correct address and they could be traced-out by Medical–registry. In this study, a newly diagnosed TB case was defined as bacteriologically (smear and culture positive report) or clinically confirmed TB patients. TB-specific deaths included TB patients in whom TB was cited as the cause of death and non-specific deaths included TB patients in whom TB was not cited as the cause of death. Comorbidities defined as patients hospitalized more than once or who visited outpatient service more than twice within a year before joining the TB registry. For all patients, dependent variable (time to TB-specific deaths) and the independent variables (sex, age, marital status, education, nationality, residency area, family size, TB type, Comorbidities) were investigated. The latest follow up (Jun 2015) for patients discharged with no death record were carried out by phone. The cause of death for those who expired check by medical –registry and by interviewing the close relatives.

This retrospective cohort study was approved by the Ethical Committee of Dr. Masih Daneshvari Hospital.

### Statistical analyses

The effect of the independent variables such as sex, age, marital status, education, nationality, residency area, family size, TB type, Comorbidities on patients’ survival was evaluated. The distance between diagnosis of TB until death (time in days) was used as survival time and patients who were still alive (until last follow-up date) considered as censored. Chi-square test was applied for testing distribution of characteristics among patients. To study the risk factors for pulmonary tuberculosis, generalized gamma regression model has been used. Moreover, to investigate the simultaneous effect of independent variables on patients’ survival all the variables in the univariate model was also entered into multivariate model. The only exception was age of start smoking variable, which had colinearity with smoking factor. In all analyses, α=0.05 was considered as the significant level and Stata 11 software was used for all analyses.

## Results

Table 1 & 2 summarize the baseline characteristics of the TB patients under study, of 2299 enrolled patients, 770 (33.4%) died and 134 (5.8%) returned as the relapses cases. The remaining had successful treatment (1395; 60.6%) with no sign of recurrence. Male to female ratio was almost similar, but the ratio of male patients in TB death cases was significantly higher than female cases. Majority of TB deaths was found in patients with higher age, between 50–69 and ≥ 70 yr (Table 1). The death rate was higher in divorced and widow patients. Patient’s educational level showed that illiterate cases had the highest rate of death (Table 1).

**Table 1:** Demographic characteristic of newly registered tuberculosis patients referred to the National Referral Center for Tuberculosis at Dr. Masih Daneshvari Hospital Tehran, Iran

***Factor***	***Category***	***Alive N(%)***	***Dead N(%)***	***P-value***
Gender	Female	835 (74.5)	286 (25.5)	< 0.001
	Male	694 (58.9)	484 (41.1)	
Age group(yr)	Under 20	75 (83.3)	15 (16.7)	< 0.001
	20–34	350 (76.6)	107 (23.4)	
	35–49	244 (65.8)	127 (34.2)	
	50–69	434 (68.0)	204 (32.0)	
	70 and older	426 (57.3)	317 (42.7)	
Marital status	Single	279 (69.9)	120 (30.1)	< 0.001
	Married	1,054 (68.0)	497 (32.0)	
	Divorced	34 (47.9)	37 (52.1)	
	Widow	162 (58.3)	116 (41.7)	
Educational level	Illiterate	650 (63.1)	380 (36.9)	< 0.001
	Primary school	333 (66.2)	170 (33.8)	
	Secondary school	252 (66.8)	125 (33.2)	
	High school	196 (73.7)	70 (26.3)	
	Higher education	98 (79.7)	25 (20.3)	
Nationality	Iranian	1,255 (64.8)	681 (35.2)	< 0.001
	Foreigners	274 (75.5)	89 (24.5)	
Residency area	Urban	1,225 (66.8)	608 (33.2)	0.553
	Rural	304 (65.4)	161 (34.6)	
Family size	Single	95 (49.0)	99 (51.0)	< 0.001
	2	390 (62.9)	230 (37.1)	
	3–5	806 (71.5)	322 (28.5)	
	More than 5	235 (67.0)	116 (33.0)	

**Table 2:** Baseline characteristics of newly registered tuberculosis patients referred to the National Referral Center for Tuberculosis at Dr. Masih Daneshvari Hospital Tehran, Iran

***Factor***	***Category***	***Alive N(%)***	***Dead N(%)***	***P-value***
Drug adverse effect *	No	705 (68.7)	321 (31.3)	< 0.001
	Yes	238 (59.8)	160 (40.2)	
Smoker	No	1,126 (72.3)	432 (27.7)	< 0.001
	Yes	395 (54.3)	333 (45.7)	
Age of start smoking (for smokers)	Under 20	142 (55.0)	116 (45.0)	0.352
	20–24	105 (48.6)	111 (51.4)	
	25–29	44 (59.5)	30 (40.5)	
	30–34	24 (55.8)	19 (44.2)	
	35–49	34 (60.7)	22 (39.3)	
	50 and older	13 (65.0)	7 (35.0)	
Passive smoker	No	1,399 (66.8)	694 (33.2)	0.426
	Yes	105 (70.0)	45 (30.0)	
Drug user	No	1,239 (70.6)	515 (29.4)	< 0.001
	Yes	285 (53.6)	247 (46.4)	
Contact with other TB patients	No	1,211 (65.1)	650 (34.9)	< 0.001
	Yes	312 (73.9)	110 (26.1)	
Imprisoned	No	1,396 (70.0)	598 (30.0)	< 0.001
	Yes	85 (43.4)	111 (56.6)	
Has Pulmonary TB	No	133 (71.5)	53 (28.5)	< 0.001
	Yes	1,393 (66.1)	716 (33.9)	
Has extra-Pulmonary TB	No	1,325 (66.4)	669 (33.6)	0.91
	Yes	201 (66.8)	100 (33.2)	
Diabetic Mellitus	No	1,241 (66.1)	637 (33.9)	0.36
	Yes	288 (68.4)	133 (31.6)	
HIV positive	No	1,472 (68.7)	670 (31.3)	< 0.001
	Yes	57 (36.3)	100 (63.7)	
Comorbidities (other than HIV or Diabetes)	No	1,126 (70.4)	473 (29.6)	< 0.001
	Yes	403 (57.7)	295 (42.3)	

Although the rate of death was higher in HIV positive people, this rate was lower in those who had diabetes mellitus or previous contact with other TB patients (Table 2). In other comorbidities i.e., cancer, liver disorders, etc, the TB deaths rate was high and significantly important (295/698; 42.3%).

From 770 TB deaths, 585 cases (75.9%) had documented “time of deaths” whereas no time was recorded, for 185 (24.02%). Majority of deaths occurred in the first year of treatment (369/585; 63.1%); while they were in hospital (250/369; 67.7%) or outside of hospital (119/369; 32.2%). The frequency was lowered from the second year (93/585; 15.90%) on words, reaches to 0.51% (3/585) in 10 yr of follow-up. Overall, the deaths rate was more in hospital (397/770; 51.5%) than outside of hospital (373/770; 48.4%), but the differences were not statistically significant. In this study, the TB –related deaths and other causes of deaths were 615 (79.8%) and 155 (20%), respectively.

The results of univariate generalized gamma regression model are presented in Table 3. The significant factors for TB deaths in univariate analysis were gender, high school education, higher education, smoker, drug user, TB contact and HIV positive (Table 3).

**Table 3:** Results of univariate generalized gamma regression model for assessing the effect different factors on patients’ survival

***Factor***	***Category***	***Generalized gamma regression model* RR (CI^†^)**
Gender	Male/Female	2* (1.1–3.7)
Age	-	1 (1–1)
Marital Status	Married/Single	0.9 (0.5–1.6)
	Widow/Single	1.5 (0.4–5.5)
	Divorced/Single	1.5 (0.7–3.2)
Education	Primary/Illiterate	1.1 (0.7–1.8)
	Secondary/Illiterate	1.3 (0.7–2.3)
	High school/Illiterate	0.3* (0.2–0.7)
	Higher education/Illiterate	0.3* (0.1–0.9)
Nationality	Iranian/Non-Iranian	0.8 (0.4–1.3)
Residency area	Rural/Urban	1.4 (0.9–2.3)
Family size	-	1 (0.9–1.1)
Adverse effect	Yes/No	1.1 (0.6–2)
Smoker	Yes/No	2.5* (1.4–4.2)
Age of start smoking	-	1 (1–1)
Passive smoker	Yes/No	1 (0.4–2.2)
Drug user	Yes/No	2.4* (1.4–4)
TB Contact	Yes/No	0.5* (0.3–0.8)
Imprisoned	Yes/No	2 (0.9–4.6)
Pulmonary TB	Yes/No	0.9 (0.4–2)
Extra-Pulmonary TB	Yes/No	0.9 (0.5–1.6)
Diabetic Mellitus	Yes/No	1 (0.6–1.7)
HIV Positive	Yes/No	4* (1.7–9.2)
Comorbidities	Yes/No	1.4 (0.8–2.3)

† 95% Confidence interval RR= Relative Risk

* *P*-value < 0.05

However, in multivariate analyses, gender, age, adverse drug effect, smoker, TB contact, diabetic mellitus, HIV positive and comorbidities were significant (Table 4). Overall, male gender, being smoker, facing drug adverse effect, drug use, having HIV positive, diabetic mellitus and comorbidities will significantly increase the risk of death and decrease the survival time.

**Table 4:** Results of multivariate generalized gamma regression model for assessing the effect different factors on patients’ survival

***Factor***	***Category***	***Generalized Gamma regression model, RR (CI^†^)***
Gender	Male/Female	5.5* (2.2–13.5)
Age	-	1.1* (1–1.1)
Marital Status	Married/Single	0.3 (0.1–1.3)
	Widow/Single	1.9 (0.2–19.3)
	Divorced/Single	1.5 (0.3–9.3)
Education	Primary/Illiterate	0.8 (0.3–2.2)
	Secondary/Illiterate	2 (0.6–6.5)
	High school/Illiterate	0.8 (0.2–3)
	Higher education/Illiterate	1 (0.1–7.2)
Residency area	Rural/Urban	1.6 (0.5–5.2)
Nationality	Foreigners/Iranian	1.8 (0.7–4.7)
Family size	-	1 (0.8–1.3)
Adverse effect	Yes/No	2.5* (1.2–5.4)
Smoker	Yes/No	3.3* (1.2–9.4)
Passive smoker	Yes/No	0.7 (0.1–3)
Drug user	Yes/No	2.1 (0.6–6.7)
TB Contact	Yes/No	0.2* (0.1–0.5)
Imprisoned	Yes/No	0.4 (0.1–2.2)
Pulmonary TB	Yes/No	1.4 (0.3–7.5)
Extra-Pulmonary TB	Yes/No	0.9 (0.2–3.3)
Diabetic Mellitus	Yes/No	3* (1–8.3)
HIV Positive	Yes/No	26* (4.6–145.9)
Comorbidities	Yes/No	4.9* (2–11.6)

† 95% Confidence interval RR= Relative Risk

* *P*-value < 0.05

## Discussion

This is the first study that evaluates the TB deaths for a rather long follow-up period in Iran. The investigation on mortality rate was not only monitored through medical registry but also by interviewing the close by relatives. The overall mortality rate was 33.4% (770/2299), which is significantly higher than previous reports (13). In total, 79.8% of deaths were related to TB specific and 20% to non–TB specific deaths. Interestingly, majority of deaths occurred in the first year of treatment (63.1%), whether in Hospital (250/369; 67.7%) or outside of Hospital (119/369; 32.2%).

The study outlines the high mortality rate under complete treatment protocols. Therefore, a considerable amount of work must be performed to achieve the goal of a TB mortality rate less than 1.0 per 100000 individuals. In this study, the associated risk factors for TB deaths were identified. The current observation shows an increased risk of deaths in male patients (484/770; 62.8%).

In previous studies male sex have been reported as an independent risk factor for TB deaths (14). They identified alcohol abuse, smoking behavior and utilization of health services as the main contribution to higher male deaths. In the present study, 65.7% of died male patients were smoker and 50.5% of them were drug user. While in died female patients the rate drops to 8.4% and 3.6%, respectively. Thereby a direct correlation between gender, smoking, drug user and TB deaths was observed. The advanced age has been reported by other investigators as risk factors in TB deaths. Likewise, both univariate and multivariate analyses showed significant importance. Although, the risk of TB death in old age (≥ 50) was more in female (77.2%) than male (62.7%). In overall, the old age was related to tuberculosis whether in alive (860/1529; 56.2%) or death cases (521/770; 67.6%). Generally, TB among the elderly is often caused by the recurrence of old infections (15). That is may account for the weakness of the immune system and/or socio-economical problem (16).

In our study, 63.7% deaths were associated with HIV. Of all risk factors for TB deaths, HIV is by far the most potent factor. In this regards, a mortality rate up to 10 times higher in HIV positive patients compared to those without positive serology of HIV (16, 17).

Several factors, such as delays in diagnosis and advanced immunosuppression, intravenous drug users were explained this poor outcome. Recently, the importance of Albumin levels and weights were underlined as an unsuccessful outcome in TB-HIV co-infection (18). Our data show that, the mortality was high among those who presented to the Hospital within the first year of treatment. This could be attributed to delay in presentation and drug adverse effect. Mortality as a delay in presentation has been already reported by many investigators.

About 56% of deaths in Nigeria were associated with delay in diagnosis due to patient’s factors (19). In Iran, about 57% of patients with TB are diagnosis, of which majority fall into late diagnosis categories (20). Delay in diagnosis is unacceptable as it may compromise the chance of a successful outcome and lead to increase transmission of TB both in household and in the community. In this study, pulmonary TB was the most common form of lesion associated with TB-deaths (716/770; 92.9%).

The other risk factors were residing in rural area, having a smoker in household, being imprisoned, and having comorbidities. Similar studies Asian countries showed association of male sex, anemia, dyspnea, chronic heart disease, malignancy, and ICU admission as a baseline prognostic factors for death during treatment of adult patients with pulmonary TB. In few studies, elderly age, Eastern residence, positive sputum bacteriology, abnormal chest X-ray and comorbidity with chronic kidney disease, stroke or chronic liver disease were most likely to be the cause of TB-specific deaths (21, 22). We demonstrated that the comorbidities (other than HIV and diabetic) have significant effect (42.3%) on TB deaths. The three most frequently comorbidities diseases were identified as high blood pressure (43.3%) ischemic heart diseases (26.3%) and hepatitis C virus (11.8%). Indeed, correlation of TB deaths with comorbidities is various in different situations have been reported (23).

Recent studies documented comorbidity with chronic kidney disease, stork, or chronic liver diseases was associated with TB specific deaths (4, 24), but in our study they were less likely to be implicated. The association of chronic kidney diseases (6.6%), stroke (2.9%) and cancer (2.0%) was not statistically significant. Usually, TB is curable diseases, and it is unlikely that a patient die from TB, until and unless there is serve or complicated chronic diseases. The high mortality rates (33.4%) highlight the urgent needs for re-evaluation of the tuberculosis control program within the country. Although, as this study performed in a referral center of tuberculosis, we receive most complicated patients throughout the country. Thereby, the collected data may be a bit higher, but still we cannot ignore the high rate of mortality, especially when the study performed in large sample size and long duration of follow-up.

Additionally, for the first time we not only take the information through medical registry, but also by interviewing the patients or their family members. To out astonished, considerable number of TB deaths cases (≥ 55) was identified when the family where interviewed. Therefore, gathering information by just reviewing the medical registry is not optional in Iran. This may explain the discrepancies between present and previous reports, and highlight the need for extensive investigation on accuracy of medical registry systems within the country.

Using generalized gamma regression model showed that variables such as gender, high school education, higher education, smoker, drug user, TB contact and HIV positive in univariate model and variables of gender, age, adverse drug effect, smoker, TB contact, diabetic mellitus, HIV positive and comorbidities in multivariate model can described survival time of TB patients from starting their treatment to death. These results are consistent with the findings of most studies (11, 12, 14, 23–26).

Moreover, more detailed analyses based on generalized gamma regression model showed that variables of male gender, age, adverse effect, smoker, drug user, diabetic mellitus, HIV positive and comorbidities will increase death hazard and reduce survival time of patients. Conversely, variables of TB contact and education (high school and higher school education) reduce death hazard and will increase survival time of patients.

## Conclusion

Despite implication of DOTS strategies over the past 15 yr, the mortality rate is high in Iran. Improvement of TB surveillance and rapid diagnosis strategies in Iran need to be emphasized. The associated risk factors for deaths are male gender, advanced age, smoking, facing drug adverse effect, drug use, having HIV positive, diabetic mellitus and comorbidities.

## Ethical considerations

Ethical issues (Including plagiarism, informed consent, misconduct, data fabrication and/or falsification, double publication and/or submission, redundancy, etc.) have been completely observed by the authors.
